# Cost-effectiveness of proton beam therapy vs. conventional radiotherapy for patients with brain tumors in Sweden: results from a non-randomized prospective multicenter study

**DOI:** 10.1186/s12962-024-00577-6

**Published:** 2024-09-13

**Authors:** Filipa Sampaio, Ulrica Langegård, Patricio Martínez de Alva, Sergio Flores, Camilla Nystrand, Per Fransson, Emma Ohlsson-Nevo, Ingrid Kristensen, Katarina Sjövall, Inna Feldman, Karin Ahlberg

**Affiliations:** 1 Department of Public Health and Caring Sciences (IFV), Uppsala University, BMC, Husargatan 3, Uppsala, 751 22 Sweden; 2 Institute of Health and Care Sciences, Sahlgrenska Academy, University of Gothenburg, Gothenburg, Sweden; 3 Department of Oncology, Sahlgrenska University Hospital, Gothenburg, Sweden; 4 Department of Nursing, Umeå University, Umeå, Sweden; 5 University Health Care Research Center, Faculty of Medicine and Health, Örebro University, Örebro, Sweden; 6 Department of Hematology, Oncology and Radiation Physics, Lund University Hospital, Lund, Sweden; 7 Department of Clinical Sciences, Oncology and Pathology, Lund University, Lund, Sweden; 8 Faculty of Health Sciences, Kristianstad University, Kristianstad, Sweden

**Keywords:** Economic evaluation, Cost-effectiveness analysis, Two-part model, Brain tumors, Proton therapy

## Abstract

**Background:**

This study assessed the cost-effectiveness of proton beam therapy (PBT) compared to conventional radiotherapy (CRT) for treating patients with brain tumors in Sweden.

**Methods:**

Data from a longitudinal non-randomized study performed between 2015 and 2020 was used, and included adult patients with brain tumors, followed during treatment and through a one-year follow-up. Clinical and demographic data were sourced from the longitudinal study and linked to Swedish national registers to get information on healthcare resource use. A cost-utility framework was used to evaluate the cost-effectiveness of PBT vs. CRT. Patients in PBT group (*n* = 310) were matched with patients in CRT group (*n* = 40) on relevant observables using propensity score matching with replacement. Costs were estimated from a healthcare perspective and included costs related to inpatient and specialized outpatient care, and prescribed medications. The health outcome was quality-adjusted life-years (QALYs), derived from the EORTC-QLQ-C30. Generalized linear models (GLM) and two-part models were used to estimate differences in costs and QALYs.

**Results:**

PBT yielded higher total costs, 14,639 US$, than CRT, 13,308 US$, with a difference of 1,372 US$ (95% CI, -4,914–7,659) over a 58 weeks’ time horizon. Further, PBT resulted in non-significantly lower QALYs, 0.746 compared to CRT, 0.774, with a difference of -0.049 (95% CI, -0.195–0.097). The probability of PBT being cost-effective was < 30% at any willingness to pay.

**Conclusions:**

These results suggest that PBT cannot be considered a cost-effective treatment for brain tumours, compared to CRT.

**Trial registration:**

Not applicable.

**Supplementary Information:**

The online version contains supplementary material available at 10.1186/s12962-024-00577-6.

## Novelty and Impact

This study informs on the cost-effectiveness of proton beam therapy (PBT) versus conventional radiotherapy (CRT) for patients with brain tumors in Sweden. Amidst global concerns regarding the cost-effectiveness of PBT, our investigation fills a notable gap by providing evidence from the Swedish healthcare landscape. Using real-world data, our study demonstrates the application of established health economic methodology to compare PBT and CRT for brain tumors, furthering research in this area.

**Supplementary Information:**

The online version contains supplementary material available at 10.1186/s12962-024-00577-6.

## Introduction

Primary brain tumors are relatively rare pathologies but cause significant morbidity and mortality among all age groups. Survival rates for brain tumors are less than 30% at 5 years after diagnosis [1]. While the burden of primary brain tumors have increased steadily since the 1990’s [2], the global prevalence of brain and central nervous system cancer was just above 1 million cases in 2019. Sweden has 3.5 times higher incidence (15.6 cases per 100,000) than the global estimate, with around 1,400 new cases of tumors diagnosed every year [3, 4]. This can be due to Sweden´s comprehensive healthcare system allowing for thorough medical examinations, which can contribute to the identification of more cases, as well as robust cancer registries and mandatory reporting systems that ensure more accurate and comprehensive recording of cancer cases, including brain tumors, compared to other countries where underreporting might occur.

The treatment of brain tumors requires expensive diagnostic and therapeutic interventions, posing a significant economic burden on patients and healthcare systems [5]. Studies related to the economic burden of this disease are scarce. The latest cost-of-illness study of brain tumors in Sweden dates back to 1996, and it reported a total cost of 201 million US$ [6] at that time (362 million US$ in 2023). More recently, estimates from Canada suggested that the total societal cost of brain tumors in 2015 amounted to more than 300 million CAN$ [7] (311 million US$ in 2023).

The main treatment for primary brain tumors is attempted surgical removal within the constraints of preserving the patient’s health. However, total tumor removal through surgery alone is seldom achieved; therefore, radiation treatment often plays an important role, particularly in high-grade gliomas [8–10]. Radiotherapy is often delivered as a photon therapy, commonly known as conventional photon therapy (CRT). CRT delivers beams of photons with high exit doses and lateral scatter on the downside, potentially damaging nearby healthy tissue. Radiotherapy using proton beams instead of photons have been signaled as a treatment alternative that may improve health outcomes for patients [11]. The main advantage of proton beam therapy (PBT) is the ability to modulate the beam range, which allows health professionals to maximize the dose deposition to the extent of the tumor [12]. This should translate into less healthy tissue affected by the radiation and, therefore, fewer complications derived from the treatment. That is why PBT has been referred to as a safe and effective treatment for intracranial tumors [13], and it appears to produce less radiation-induced side effects than CRT [14], thus improving health-related quality of life (HRQoL) for the patients. However, despite the promising benefits of PBT, clinical evidence of superiority against CRT is not clear [15].

The symptoms associated with this pathology range from fatigue, sleep disturbance, and altered mood states to problems with vision, motor function, speech, pain, memory loss, confusion, and seizures [16]. These symptoms and complications may affect HRQoL in patients [17]. PBT and CRT therapies have been contrasted in several studies to explore changes in quality of life (QoL) outcomes, with results pointing to improved QoL in patients receiving PBT [18–20].

The introduction of PBT clinics globally has also raised questions of efficiency due to the heavy investments in such clinics. There are a number of cost-effectiveness analyses, mainly modelling studies, that have yielded controversial results in the evaluation of treatments for cancers e.g. prostate cancer [21]; some studies reported favorable cost-effectiveness ratios, whereas others did not. The results were highly variable and dependent on the assumptions and methodologies used in the models [22]. These studies have been criticized for uncertainties in effectiveness estimates and limitations in capturing the true costs of PBT facilities [22]. Moreover, outcome measures vary widely (e.g., overall survival, progression-free survival, HRQoL), with a notable lack of studies using quality-adjusted life years (QALYs). There are few studies looking at the cost-effectiveness of PBT specific to brain tumors, and most have evaluated pediatric medulloblastoma. In four out of five studies, PBT was reported to be cost-effective [23–27]. No economic evaluation of PBT for brain tumors in an adult population in the Swedish setting has been conducted. Economic evaluations provide useful information to identify and compare the costs and effects of alternative therapies, helping decision makers to allocate scarce resources effectively [28].

This study is part of the ProtonCare project with the overall purpose to evaluate PBT from the patients’ perspective by assessing patient-reported outcomes and experiences in patients undergoing PBT or CRT [29] (Research Ethics Committee in Gothenburg, Sweden, approval reference: Dnr:433–15). The Skandion Clinic (in Uppsala, Sweden) has been affiliated with this study since 2015, and has collected data on patients’ symptoms, side effects, and HRQoL.

The aim of this study was to estimate the cost-effectiveness of PBT compared with CRT for patients with brain tumors.

## Methods

### Overview

In Sweden, a non-randomized non-blind longitudinal study from the ProtonCare project collected data of 350 patients, which were followed during their treatment for a brain tumor, and through a one-year follow-up period [30]. Of those, 310 patients recieved PBT, while the remaining 40 received CRT. Both treatment groups received 6 weeks of the corresponding radiotherapy. Data including sociodemographic characteristics of patients, self-reported symptoms and HRQoL were collected within the trial at baseline (pre-treatment, week 0), mid-treatment (3 weeks post-baseline) and end of treatment (6 weeks post-baseline). Follow-up data was collected at 1, 3, 6, 9, and 12 months following treatment completion, totaling 58 weeks. Trial data was linked to register data on the use of healthcare resources during the 58 weeks. Further trial details are available elsewhere [30].

#### Health outcomes

The primary outcome in this economic evaluation was the QALY, a composite measure encompassing both HRQoL and mortality (length of life). HRQoL was assessed by the European Organisation for Research and Treatment of Cancer (EORTC) QLQ-C30 questionnaire, an instrument designed to measure HRQoL in patients diagnosed with cancer [31]. It includes five functional scales, three symptom scales, five single item symptoms, and a global QoL scale. All scales and single items are linearly transformed to scores ranging from 0 to 100. For functional scales and global QoL, a higher score suggests better level of functioning; for symptom scales, a higher score suggest worse symptoms [32]. As the EORTC QLQ-C30 is not a preference-based instrument, its scores cannot be directly used to calculate QALY. Therefore, the Quality of Life Utility-10 Dimensions (QLU-10D) instrument was used, which allows data collected with the EORTC QLQ-C30 to be used for the calculation of cancer-specific health utilities by providing a preference-based scoring algorithm [33]. The algorithm requires country-specific preference weights to estimate the QLU-C10D utility scores. There are currently no value sets for Sweden. For this study, we used Danish utility weights [34]. Total QALY over the study period (58 weeks) was then calculated using the area under the curve method [35] using the QLU-C10D utility scores.

#### Cost data collection

##### Costing perspective

Costs were estimated from a healthcare perspective, including patients’ use of healthcare resources and prescribed medication. Individual-level data were collected from Swedish national registers for the study period between 2015 and 2021 (on an individual level, from treatment start to one-year following treatment completion).

##### Inpatient and specialized outpatient care costs

Inpatient care stays and specialized outpatient care visits were sourced from the Swedish National Patient Register, held by the Swedish National Board of Health and Welfare. It covers approximately 93% of all somatic inpatient care in Sweden and approximately 74% of all somatic outpatient specialised care [36]. Reasons for admissions and visits were coded according to the International Classification of Disease (ICD) and Diagnosis Related Group (DRG) codes, which are used for reimbursement of hospital production. The payment formula is based on a base rate multiplied by a relative cost weight specific for each DRG. Cases within the same DRG code group are expected to have a similar clinical evolution. The costs for inpatient care stays and specialized outpatient care visits were calculated using a bottom-up approach, by multiplying each inpatient and outpatient care episode by its respective cost. Each episode of care has an assigned DRG code, which is a specific identifier used to classify hospital cases into one of the groups established by the DRG system. The code represents a set of conditions and treatments that are expected to require a similar amount of hospital resources. Each DRG code has a corresponding DRG weight in a given year. The weight reflects the relative cost of treating patients in that DRG compared to the average cost of treating all patients, and it is used to determine hospital reimbursement. Each DRG weight for each episode of care was then multiplied by the average monetary value per 1 DRG unit for the year the episode of care took place to calculate the cost of the episode. DRG weights were sourced from the National Board of Health and Welfare in Sweden for the respective years. The following formula was used:

DRG cost = DRG weight x average monetary value per 1 DRG unit.

##### Prescribed medication costs

Prescribed medication costs were collected from the Prescribed Drug Register, held by the Swedish National Board of Health and Welfare. It covers information on all prescribed drugs across all pharmacies in Sweden. The register includes information on the Anatomical Therapeutic Classification (ATC) code of each drug, amount dispensed, dosage, total cost and date of dispensing [37]. For this estimate we used the variable total costs.

##### Total costs

Costs related to individual radiotherapy sessions could not be identified in the dataset, and consequently, the costs of each PBT and CRT session could not be tracked separately. Due to this limitation, all costs were aggregated over the study period to estimate total costs without distinguishing between treatment costs and other costs related to the use of other healthcare resources. Healthcare resources were costed in 2021 Swedish Krona (SEK) and converted to 2023 U.S. dollars (US$) using Purchasing Power Parities for Gross Domestic Product [38].

### Statistical analyses

#### Missing data

The study had up to 21% of missing values regarding the EORTC QLQ-C30 items. Therefore, multiple imputation using chained equations (MICE) was performed to adress missing data, which is an appropriate imputation method even when dealing with substantial missing data [39, 40]. Under the assumption that data was likely to be missing at random (MAR), meaning that the probability of a missing value depends only on observed values and not on unobserved values [41], data was imputed in 20 datasets, and estimates were combined to obtain a pooled estimate [42]. A graphical representation of the missing data is available in the supplemental material (figures A1 to A7).

#### Matching

Since the study was a non-randomized and non-blinded study, treatment groups had unequal sample size (310 in PBT, 40 in CRT) with unbalanced background variables, making direct comparisons of costs and effects between groups unviable. To reduce the effects of selection bias, propensity score matching (PSM) was performed to match patients from both treatment groups based on background variables, aiming to make groups comparable [43]. We employed the nearest neighbour matching with the replacement method (0.15 caliper), allowing CRT patients to match with multiple PBT patients. Matching with replacement is particularly helpful in settings where the control group has fewer individuals than the treated [44, 45]. Variables used for the matching were age, sex, civil status, education, employment, type of tumor (benign or malignant), and pre-treatment depression. Depression was identified by the Hospital Anxiety and Depression Scale (HADS) [46]. Balance diagnostics and a plot of standardized mean differences of covariates between groups were used to assess matching quality. An adequate balance is generally indicated by standardized mean differences of less than 0.2 [47].

#### Analysis of cost and outcome data

Due to the generally skewed nature of healthcare utilization data, characterized in this sample by a significant proportion of zero healthcare usage (70.6% for inpatient care, 16.1% for medication), a two-part regression model was employed to analyse cost differences between PBT and CRT. This approach is suitable when data violate normality assumptions [48]. The first part of the model used a probit model for predicting the probability of any healthcare usage (and therefore costs). The second part employed a generalized linear model (GLM) for estimating the mean costs, if any, conditional on being greater than zero. A specific distribution with a log link function was used in the GLM to model the skewed distribution of the data [48]. The best distribution for each model was chosen using a Park test [49] and the suitable link function confirmed using the Akaike information criterion (AIC) to assess model fit. The two-part model was only used for medication and inpatient care costs; for outpatient care and total healthcare costs, a regular GLM was used to investigate the difference in cost between treatment arms, given that these variables had no proportion of zero costs. For analysing QALYs, a GLM with a binomial distribution to fit the skewed data bounded between 0 and 1 and a logit link function were used to estimate the difference in mean QALY between treatment groups over time (see Figures A9 and A10 in the Supplementary appendix for the distribution of QALYs in the full sample and by treatment group, and Table A4 for information on the family distributions and link functions chosen for the analysis of cost and QALY data).

#### Cost-effectiveness analysis

A cost-utility framework was employed, using QALYs as the health outcome and results expressed as cost per QALY gained. The uncertainty around the incremental cost and outcome estimates was represented on a cost-effectiveness plane using non-parametric bootstrapping with 5,000 iterations. Net monetary benefits at different thresholds of willingness to pay were calculated and presented on a cost-effectiveness acceptability curve (CEAC). The CEAC captures decision uncertainty and shows the probability of PBT being cost-effective at different willingness to pay thresholds. The CEAC is available in the supplemental material, figure A9.

#### Sensitivity analyses

Sensitivity analyses were conducted to assess the impact of different assumptions on the study results. We performed the following analyses: (1) Estimating QALYs using an algorithm developed by Versteegh et al. [50] to map EORTC QLQ-C30 scores to EQ-5D-3L utilities; (2) Estimating QALYs using an algorithm developed by McKenzie and van der Pol [51] to map EORTC QLQ-C30 scores to EQ-5D-3L utilities; (3) using Dutch tariffs [52] to estimate the QLU-C10D utility scores; (4) using German tariffs [53] to estimate the QLU-C10D utility scores; and (5) Complete case analysis, considering trial participants with complete data only at all measurement timepoints.

## Results

### Patient characteristics

Descriptive statistics of the original dataset are presented in the supplemental material, Table A1. After PSM, background variables were balanced betweeen treatment groups. Standardized mean difference was 0.0017, with 38 patients from CRT matched with 173 from PBT. Balance diagnostics and a graphical representation of the matching performed are included in supplemental material (Table A2-3, fig. A8). Descriptive statistics of background variables between treatment groups after matching are shown in Table 1.

**Table 1:** Patient characteristics by treatment groups, after matching

Variables	PBT, *n* (%)	CRT, *n* (%)
**N**	173	38
**Age**,** years**		
Mean	55	57
SD	13	13
Min	19	29
Max	80	77
**Sex**		
Male	78 (45)	19 (50)
Female	95 (55)	19 (50)
**Civil status**		
Married	138 (80)	32 (84)
Single	35 (20)	6 (16)
**Education**		
Elementary	23 (13)	6 (16)
High-school	99 (57)	22 (58)
University	51 (29)	10 (26)
**Employment**		
Employed / student	125 (72)	26 (68)
Not employed / retired	48 (28)	12 (32)
**Tumor type**		
Malignant	89 (51)	16 (42)
Benign	84 (49)	22 (58)
Depression*		
No	69 (40)	14 (37)
Yes	104 (60)	24 (63)

*Abbreviations* PBT – Proton beam therapy, CRT – Conventional radiotherapy, SD – Standard deviation *Identified by the hospital anxiety and depression scale (HADS)

The matched sample had similar distribution of background variables. The CRT group had higher mean age. The proportion of females was 5% higher in PBT. Most participants were married, their highest degree were high-school education, were employed/studying and had pre-treatment depression identified in the HADS.

### Cost and health outcomes

Results of the two-part regressions and GLMs are presented in Table 2. While PBT was associated with higher medication and inpatient care costs compared to CRT, and lower outpatient costs, these differences were not statistically significant.

**Table 2:** Results from two-part regressions and GLMs for costs (2023 US$)

	PBT		CRT			
	First part	Second part	First part	Second part	Difference in cost over 58 weeks	
Parameters	% positive expenditure, mean (95% CI)	Cost, mean (95% CI)	% positive expenditure, mean (95% CI)	Cost, mean (95% CI)	Cost, mean (95% CI)	p-value
Medication costs	0.85 (0.80–0.90)	3,206 (1,519–4,893)	0.79 (0.66–0.92)	2,686 (-451–5,825)	528 (-3,453–4,510)	0.795
Inpatient care costs	0.29 (0.22–0.36)	5,087 (3,005–7,168)	0.32 (0.17–0.46)	3,176 (968–5,393)	2,227 (-1,986–6,440)	0.330
Outpatient care costs	NA	6,346 (5,634–7,058)	NA	7,445 (5,926–8,964)	-1,099 (-2,777–578)	0.199
Total healthcare costs	NA	14,639 (11,829 − 17,449)	NA	13,308 (8,111 − 18,505)	1,372 (-4,914–7,659)	0.669

*Abbreviations* PBT – Proton beam therapy, CRT– Conventional radiotherapy

On average, PBT yielded larger total healthcare costs, 14,639 US$, compared to CRT, 13,308 US$. The difference in costs between both treatment groups was 1,372 US$ (95% CI, -4,914–7,659), not statistically significant.

In terms of outcomes, there was an increase over time in HRQoL measured with the EORTC QLQ-C30 and a decrease of QLU-C10D estimated utilities in the CRT group. In the PBT group, both estimates decreased (Table 3 and Figures A11 and A12 in the Supplementary appendix). In terms of QALYs, PBT yielded lower average QALYs, 0.746, than CRT, 0.774, with a difference of − 0.049 (95% CI: -0.195 – -0.097) QALYs, non statistically significant, with a p value 0.511.

**Table 3:** Imputed EORTC QLQ-C30 HRQoL scores and estimated QLU-C10D utilities using Danish tariffs

	Baseline	Mid	End	1 m	3 m	6 m	9 m	12 m
**CRT**								
EORTC QLQ-C30 Mean (SD)	58.9 (23.2)	53.2 (24.3)	54.3 (22.0)	62.9 (20.4)	60.9 (19.9)	61.8 (22.1)	62.9 (23.7)	63.5 (23.4)
QLU-C10D Mean (SD)	0.71 (0.19)	0.65 (0.21)	0.65 (0.21)	0.73 (0.19)	0.71 (0.20)	0.72 (0.22)	0.70 (0.26)	0.68 (0.26)
**PBT**								
EORTC QLQ-C30 Mean (SD)	67.9 (19.7)	63.9 (20.6)	61.2 (22.1)	59.6 (24.0)	62.5 (23.1)	60.3 (25.0)	63.5 (23.7)	60.7 (24.2)
QLU-C10D Mean (SD)	0.73 (0.19)	0.71 (0.19)	0.68 (0.22)	0.68 (0.22)	0.67 (0.23)	0.67 (0.23)	0.67 (0.24)	0.66 (0.25)

*Abbreviations* PBT – Proton beam therapy, CRT – Conventional radiotherapy, m – months, SD – Standard deviation

### Cost-effectiveness analysis

With incremental higher cost, 1,372 US$, and lower QALYs, -0.049, PBT was dominated by CRT (Table 4).

**Table 4:** Cost-effectiveness results (costs in 2023 US$)

	Costs mean (95% CI)	QALY mean (95% CI)	Incremental costs mean (95% CI)	Incremental QALY mean (95% CI)	ICER
PBT	14,639 (11,829 − 17,449)	0.746 (0.685–0.8074)	1,372 (-4,914–7,659)	-0.049 (-0.195–0.097)	Dominated
CRT	13,308 (8,111–18,505)	0.774 (0.648–0.901)			

*Abbreviations* PBT – Proton beam therapy, CRT– Conventional radiotherapy, ICER – Incremental cost effectiveness ratio, QALY – Quality adjusted life year

Dominated means that PBT is more costly and yields less QALYs than CRT

**Fig. 1:**
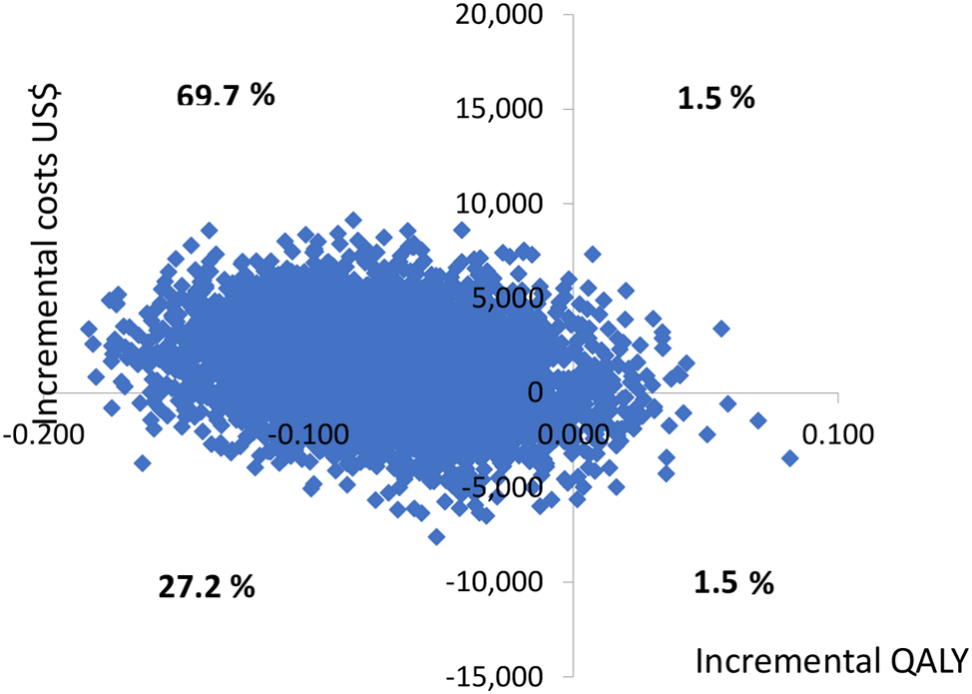
Cost Effectiveness plane PBT vs. CRT

The incremental cost-effectiveness ratio (ICER) was calculated using non-parametric bootstrapping. The results were plotted on a cost-effectiveness plane and shown in Fig. 1.

The cost-effectiveness plane illustrates the uncertainty and variability surrounding the cost-effectiveness estimates of PBT in comparison to CRT. This is visualized as a cloud of points on the plane, reflecting the 5,000 iterations of incremental costs and QALYs from the bootstrapping. On the x-axis, the difference in QALYs between interventions is presented, while the y-axis displays the difference in costs. Each point on the plane signifies a potential ICER, and its position indicates various interpretations.

Points in the north-east (NE) quadrant indicate instances where PBT results in higher QALYs at a greater cost than CRT. On the south-east (SE) quadrant, PBT demonstrates higher QALYs at a lower cost. Conversely, the north-west (NW) quadrant represents scenarios where there are fewer QALYs at a higher cost for PBT, and the south-west (SW) quadrant suggests that PBT has both fewer QALYs and lower costs than CRT.

The wide dispersion of points across west quadrants highlights the substantial uncertainty around QALY estimates. PBT yields less QALYs and higher costs than CRT in 69.7% of the iterations (NW quadrant). 27.2% of iterations appear in the SW quardant, and the remaining are distributed equally across the NE and SE quadrants.

**Fig. 2:**
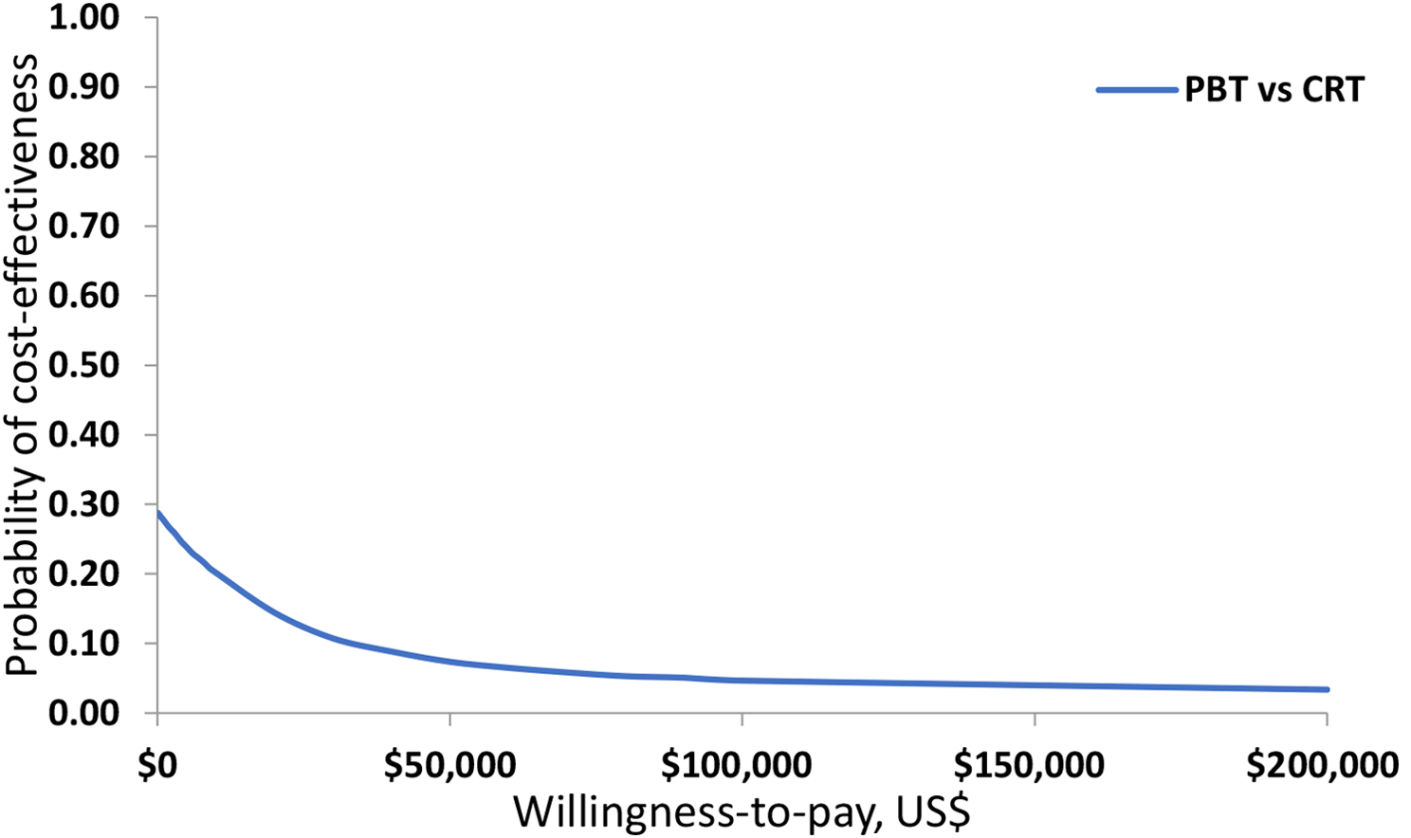
Cost-effectiveness acceptability curve

The cost-effectiveness acceptability curve (CEAC) displayed in Fig. 2 shows that at any given willingness to pay threshold, the probability of PBT being cost-effective compared to CRT is less than 30%.

### Sensitivity analyses

Table 5 shows the results of the sensitivity analyses. In the first and second scenarios, the impact of alternative mapping algorithms for calculating QALYs was explored. Using different algorithms yielded the same results as the base case, where PBT was dominated by CRT. In the third and fourth scenarios, results were robust to using different tariffs to estimate utilities. In the fifth scenario, a complete case analysis was conducted considering participants with complete data only. The difference in QALYs between arms was reduced but remained in favour of CRT and non-statistically significant.

**Table 5:** Results of the sensitivity analyses

Scenario	Incremental cost mean (95% CI)	Incremental QALY mean (95% CI)	ICER mean (95% CI)
Base case	1,372 (-4,914–7,659)	-0.049 (-0.195–0.097)	Dominated
1. Versteegh et al. mapping algorithm (50)	1,372 (-4,914–7,659)	-0.023 (-0.186–0.140)	Dominated
2. McKenzie and van der Pol mapping algorithm (51)	1,372 (-4,914–7,659)	-0.034 (-0.18–0.113)	Dominated
3. Dutch tariffs to estimate QLU-C10D utility scores	1,372 (-4,914–7,659)	-0.045 (-0.176–0.865)	Dominated
4. German tariffs to estimate the QLU-C10D utility scores	1,372 (-4,914–7,659)	-0.046 (-0.180–0.089)	Dominated
5. Complete case analysis	1,372 (-4,914–7,659)	-0.003 (-0.116–0.109)	Dominated

*Abbreviations* PBT – Proton beam therapy, CI – Confidence interval, CRT– Conventional radiotherapy, ICER – Incremental cost effectiveness ratio, QALY – Quality adjusted life year. Costs in 2023 US$

## Discussion

### Main results

This study investigated the cost-effectiveness of PBT against CRT from a healthcare perspective. The results show that PBT yielded higher healthcare costs and lower QALYs than CRT, although these differences were not statistically significant. There was large uncertainty around the cost and QALYs estimates, with PBT having less than 30% probability of being cost-effective, at any given willingness to pay. In addition, different scenarios were assessed where alternative mapping algorithms were employed compared to the base case, other tariffs were used to estimate utilities, and a complete case analysis was performed. Results were robust to changes in assumptions.

The existing literature on the cost-effectiveness of PBT for cancer treatment in Sweden remains limited. Two earlier cost-effectiveness studies examined PBT for childhood medulloblastoma and breast cancer [27, 54]. For treating medulloblastoma in children, Lundkvist concluded that PBT was cost-effective [27]. In terms of treating breast cancer with PBT, Lundkvist reported a cost per QALY gained of €65,000 in 2002 (115,717 US$ in 2023), and concluded that PBT could be cost-effective if appropriate high risk patient groups were targeted for treatment [54]. These differences in results across studies about the cost-effectiveness of PBT have been seen in the literature [55], making it hard to reach a conclusion.

In the most recent systematic literature review on cost-effectiveness studies of PBT, 18 international studies were included from the period 2000–2015 for different types of cancer [56]. The review showed that PBT was cost-effective for certain cancers, including pediatric brain tumors, well-selected breast cancers, locally advanced lung cancer, and high-risk head/neck cancers, but not for prostate cancer or early-stage lung cancer. The review discussed that careful patient selection is crucial for assessing its cost-effectiveness and concluded that PBT might not be the most economical option for all cancers or patients within a specific cancer type.

### Strengths and limitations

There are some limiting factors that should be considered when interpreting the results of this study. The main limitation was the restricted costing perspective. The costs included in the analysis were estimated from a healthcare perspective, encompassing patients’ utilization of healthcare services and prescribed medication. Unfortunately, data limitations precluded the incorporation of costs related to individual radiotherapy sessions, capital and operational costs across treatment cohorts. As a result, we could not separately track the costs of each PBT and CRT session, hence costs were aggregated over time. A detailed costing analysis including the cost per radiotherapy session would be crucial for accurate cost representation, and give a better insight into the cost implications of the PBT therapy. Future studies should address this limitation. This limitation mirrors a common challenge noted in the existing literature, where the absence of comprehensive data on both costs and outcomes introduces potential biases into the evaluation of the cost-effectiveness of PBT [22].

Furthermore, a mapping technique was used to estimate QALYs in this economic evaluation. Although we employed the official algorithm from EORTC QLQ-C30 to estimate QLU-C10D utilities, variations in the trajectories of the quality of life estimates were observed. The algorithm for conversion of EORTC QLQ-C30 scores to QLU-C10D utilities uses only 13 items out of the 30 EORTC QLQ-C30 items, and 10 domains out of the total 15 domains, and misses out on the following domains: Cognitive functioning, Social functioning, Dyspnea, Financial difficulties, and Global health status/QoL. This conversion process might emphasize different aspects of health and well-being, possibly underestimating improvements captured by the broader EORTC QLQ-C30 instrument. However, instrument mapping is a well established solution when preference-based data is unavailable [57].

Another limiting factor was the cohort size and unbalanced composition of the treatment groups, which was reduced when matching participants based on background variables between treatment groups. The reason for this imbalance in the groups was difficulties in recruiting patients to the CRT group. Regardless of using PSM with replacement as a technique to address the challenges of analysing observational studies due to covariate imbalance [58], the participant count in the CRT group was low from the start. Using PSM with replacement, where participants may be used as matches multiple times, may lead to increased variability and uncertainty in the CRT estimates. Importantly, given the small sample size, it is plausible that the study was underpowered to detect statistically significant differences. Future studies with larger sample sizes and a priori power calculations are needed to confirm our findings.

These analyses are also restricted to a one-year time horizon, which limits the conclusions that can be drawn on the health and economic impacts of these two treatments to the short term. Often, trials do not follow participants over a sufficiently long period to be able to determine the sustainability of treatment effects. In our study, this limitation was mitigated by linking trial data with national register data on inpatient, outpatient and medication, albeit still limited to a one-year follow-up post treatment. National and international health technology assessment agencies recommend a lifetime horizon when evaluating the cost-effectiveness of new treatments. This requires good data sources for patients over a longer time period or other methodological approaches, such as decision modelling, to be able to extrapolate the health and economic outcomes of these two treatments. Data on the longer-term impacts of these treatments beyond one year could yield different results than the ones observed in this short term study. Further studies are encouraged to consider these aspects.

Despite these limitations, our study demonstrates the application of established health economics methodology to compare PBT and CRT for brain tumors, furthering research in this area, highlighting the value of linking trial data with national routinely collected register data. Studies directly collecting cost and effect data are warranted to better assess the value of PBT in the Swedish brain tumor treatment landscape.

## Electronic supplementary material

Below is the link to the electronic supplementary material.

Supplementary Material 1

## Data Availability

Data may be available upon reasonable request and subject to ethical and privacy considerations. Please contact the corresponding author for inquiries.

## Abbreviations

CEAC: Cost-effective acceptability curve
CRT: Conventional radiotherapy
DALY: Disability-adjusted life year
DRG: Diagnosis related group
EORTC: European Organization for Research and Treatment of Cancer
GLM: Generalized linear model
HADS: Hospital anxiety and depression scale
HRQoL: Health-related quality of life
ICER: Incremental cost-effectivenes ratio
MAR: Missing at random
MICE: Multiple imputation by chained equations
PSM: Propensity score matching
PBT: Proton beam therapy
QALY: Quality-adjusted life year
QoL: Quality of life
SEK: Swedish krona
