# Supplementary material for: Cost-effectiveness of proton beam therapy vs. conventional radiotherapy for patients with brain tumors in Sweden: results from a non-randomized prospective multicenter study
Source: Cost Eff Resour Alloc. 2024 Sep 13;22:66. doi: 10.1186/s12962-024-00577-6 (PMC11396687; doi:10.1186/s12962-024-00577-6)
Supplement: Supplementary file 1 — Supplementary Material 1 [file 12962_2024_577_MOESM1_ESM.docx]

| Cost-effectiveness of proton beam therapy vs conventional radiotherapy for patients with brain tumours in Sweden: results from a non-randomized prospective multicenter study |
| --- |
| Supplementary material |
| Filipa Sampaio PhD, Ulrica Langegård PhD RN, Patricio Martínez de Alva MD MSc, Sergio Flores MD MSc, Camilla Nystrand PhD, Per Fransson Professor RN, Emma Ohlsson-Nevo PhD RN, Ingrid Kristensen PhD RN, Katarina Sjövall PhD RN, Inna Feldman PhD, Karin Ahlberg PhD RN |

|  |
| --- |

Contents

[Missing data pattern of EORTC QLQC30 scores at all data collection points 2](#_Toc174914056)

[Descriptive statistics of trial sample before matching 4](#_Toc174914057)

[Balance diagnostics of propensity score matching 5](#_Toc174914058)

[Distribution of QALYs after imputation and after matching 6](#_Toc174914059)

[Family distributions and link functions chosen for analysis of cost and QALY data 7](#_Toc174914060)

[Distribution of scores of EORTC QLQ-C30 and QLU-C10D over the study period by treatment group (after imputation and after matching) 7](#_Toc174914061)

# Missing data pattern of EORTC QLQC30 scores at all data collection points

Figure A1. Missing data pattern of EORTC QLQC30 scores at mid-treatment period.


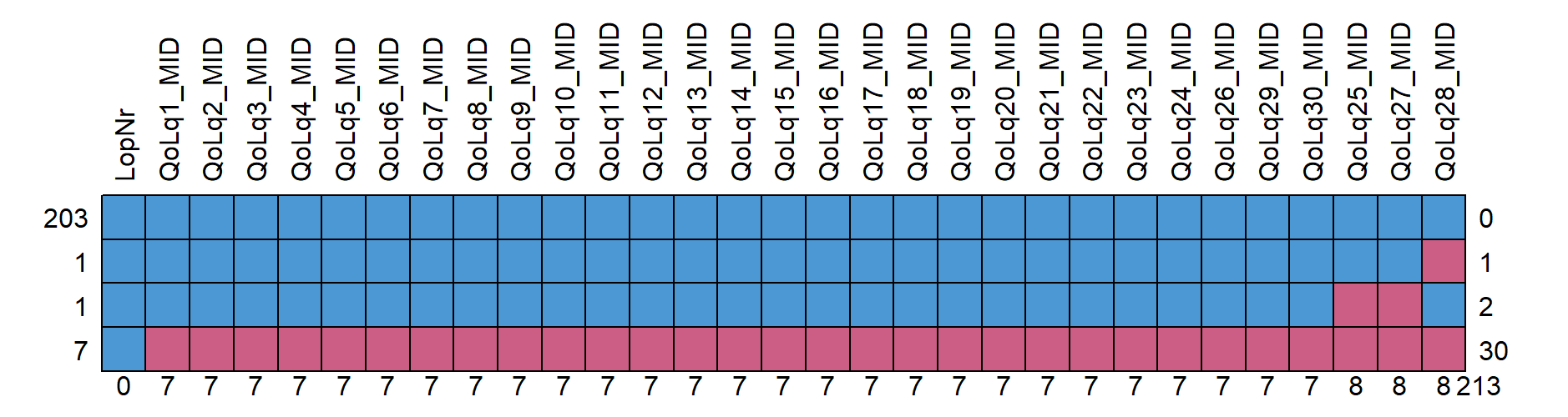


Figure A2. Missing data pattern of EORTC QLQC30 scores at end-treatment period.


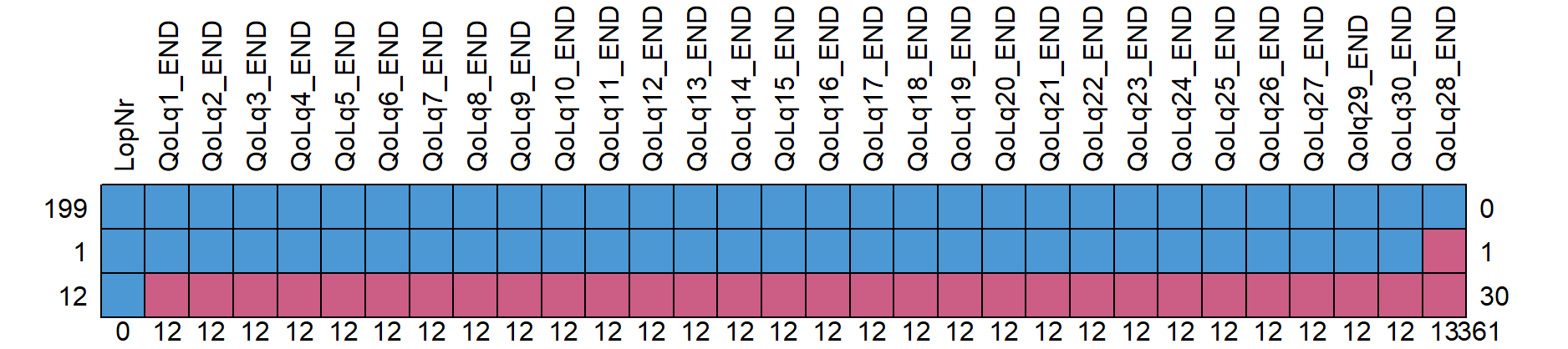


Figure A3. Missing data pattern of EORTC QLQC30 scores at one-month follow-up period.


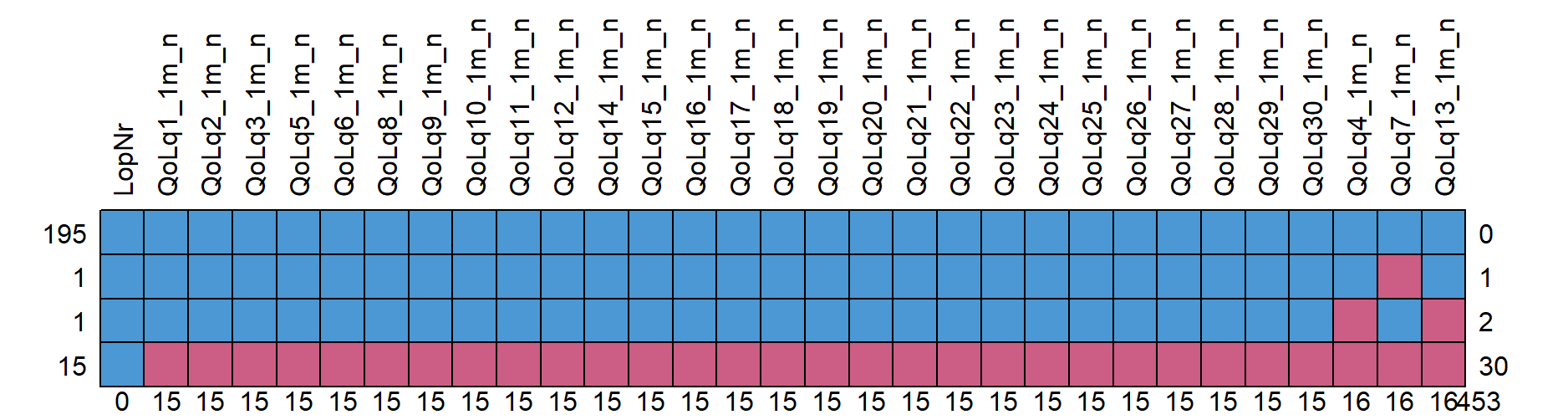


Figure A4. Missing data pattern of EORTC QLQC30 scores at 3-month follow-up period.


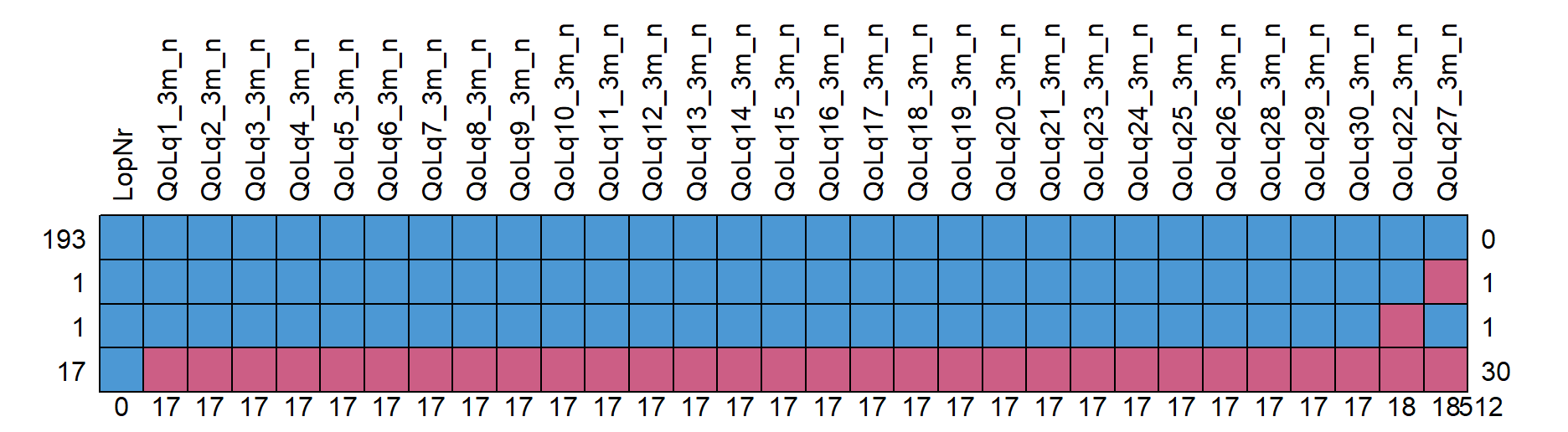


Figure A5. Missing data pattern of EORTC QLQC30 scores at 6-month follow-up period.


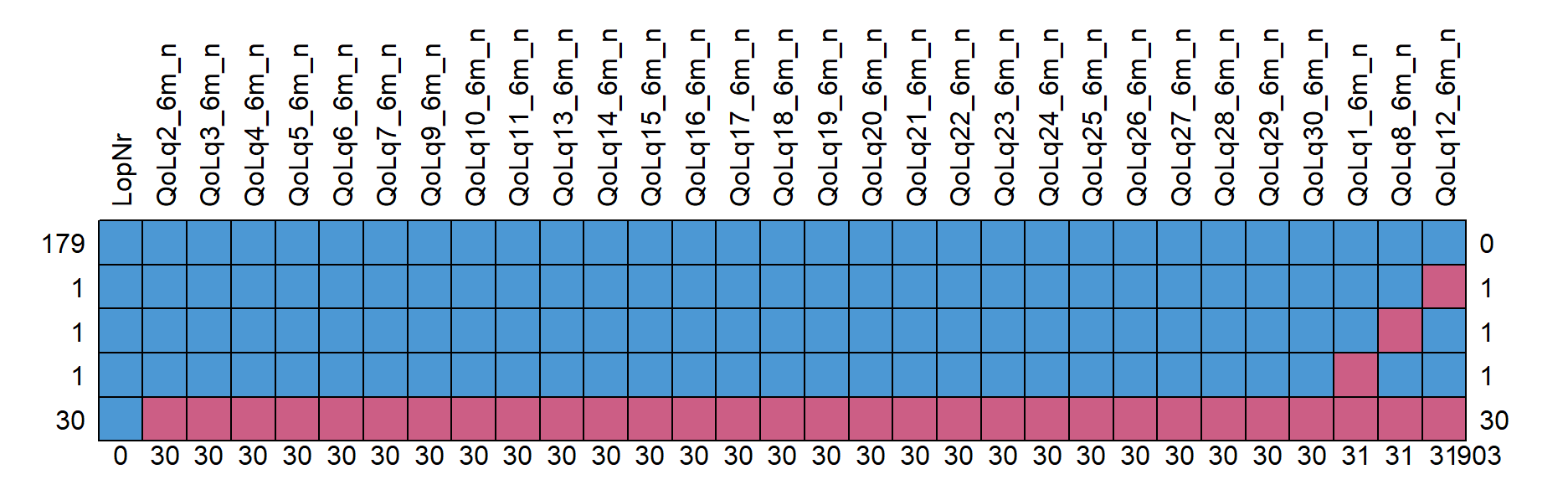


Figure A6. Missing data pattern of EORTC QLQC30 scores at 9-month follow-up period.


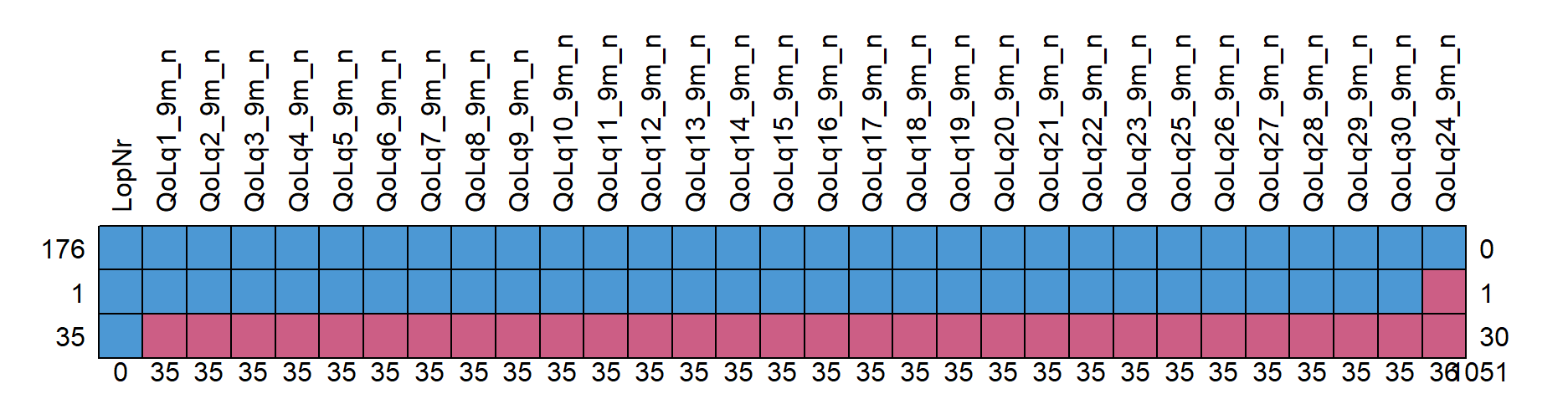


Figure A7. Missing data pattern of EORTC QLQC30 scores at 12-month follow-up period.


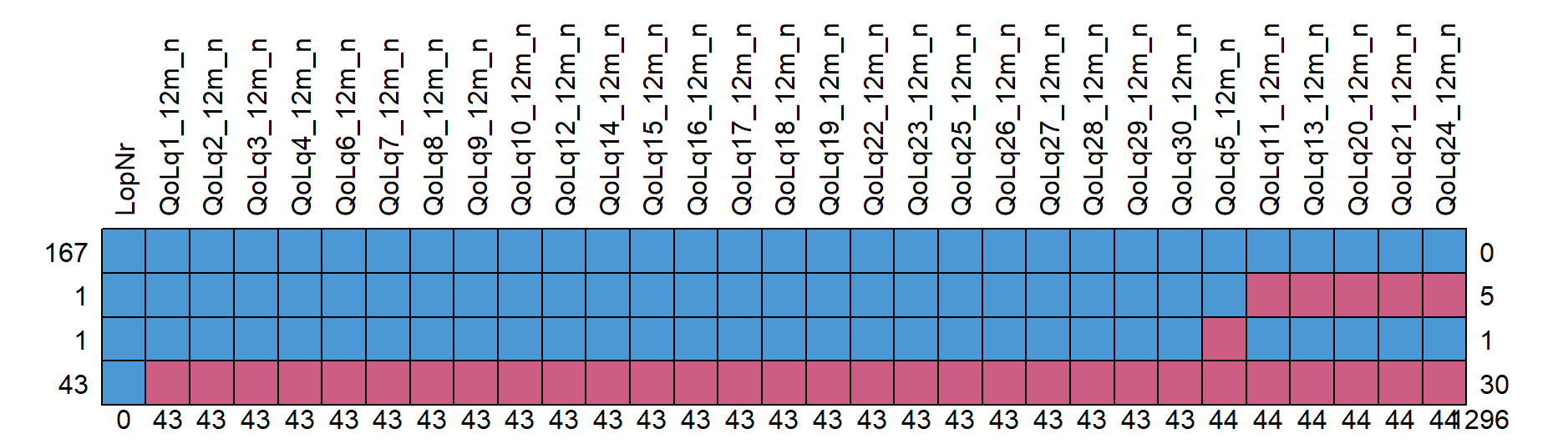


*Each row represents a pattern. Blue squares are non-missing variables, red squares represent missing values. Left numbers in each pattern table show the number of times that specific pattern (row) was identified in the data. Numbers to the right of the table specify the number of missing values in each pattern. Numbers below the table represent the number of missing values of each variable.

# Descriptive statistics of trial sample before matching

Table A1. Demographics of trial sample before matching.

| **Variables** | PBT, n (%) | CRT, n (%) |
| --- | --- | --- |
| **Age** |  |  |
| Mean | 49 | 58 |
| Sd | 14 | 12.8 |
| Min | 19 | 29 |
| Max | 80 | 77 |
| **Sex** |  |  |
| Male | 149 (47.1) | 19 (46.3) |
| Female | 167 (52.8) | 22 (53.6) |
| **Civil status** |  |  |
| Married | 224 (70.8) | 35 (85.3) |
| Single | 92 (29.1) | 6 (14.6) |
| **Education** |  |  |
| Elementary | 31 (9.9) | 7 (17.9) |
| Highschool | 142 (45.5) | 22 (56.4) |
| University | 139 (44.5) | 10 (25.6) |
| **Employment** |  |  |
| Employed / student | 255 (81.2) | 26 (63.4) |
| Not employed / pensioner | 59 (18.7) | 15 (36.5) |
| **Tumor type** |  |  |
| Malign | 183 (57.9) | 16 (40) |
| Benign | 133 (42) | 24 (60) |
| **Depression** |  |  |
| Yes | 33 (10.6) | 8 (19.5) |
| No | 276 (89.3) | 33 (80.4) |

Abbreviations: PBT – Proton beam therapy, CRT – Conventional radiotherapy.

# Balance diagnostics of propensity score matching

Table A2. Propensity score test of matching variables.

| Variable | Treated | Control | %bias | t | p>t | V(T)/V(C) |
| --- | --- | --- | --- | --- | --- | --- |
| Age | 54.55 | 53.12 | 11.2 | 1.00 | 0.31 | 0.94 |
| Sex | 1.54 | 1.55 | -1.1 | -0.11 | 0.91 | 1.00 |
| Civil status | 1.20 | 1.15 | 12.0 | 1.12 | 0.26 | 1.23 |
| Education | 2.16 | 2.27 | -18 | -1.73 | 0.08 | 1.11 |
| Employment | 1.27 | 1.23 | 10.0 | 0.99 | 0.32 | 1.13 |
| Tumor type | 1.48 | 1.51 | -5.8 | -0.54 | 0.59 | 1.0 |
| Depression | 0.60 | 0.65 | -11.8 | -1.11 | 0.26 | . |

Table A3. Overall measures of covariate imbalance.

| Ps R2 | LR chi2 | p>chi2 | MeanBias | MedBias | B | R | %Var |
| --- | --- | --- | --- | --- | --- | --- | --- |
| 0.011 | 5.10 | 0.648 | 10.0 | 11.2 | 24.4 | 0.95 | 0 |

Figure A8. Propensity score density before and after matching.


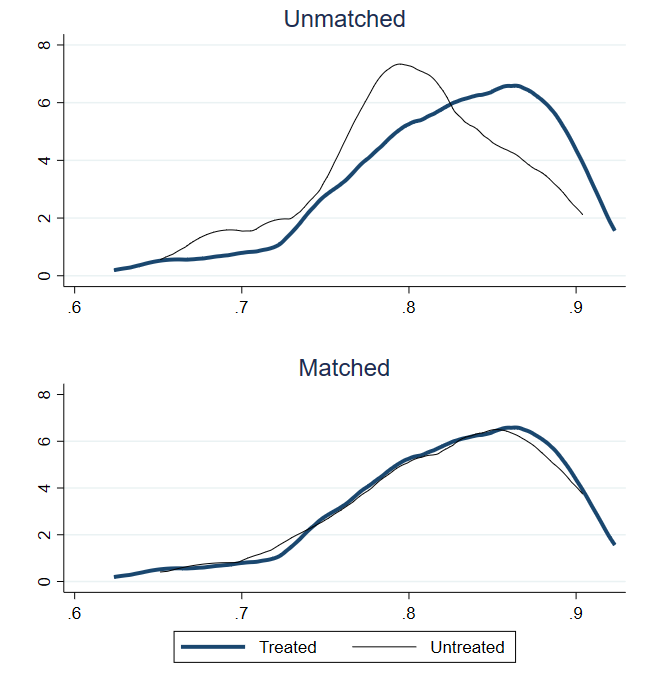


# Distribution of QALYs after imputation and after matching

Figure A9. Distribution of QALYs after imputation and after matching in the full sample

**
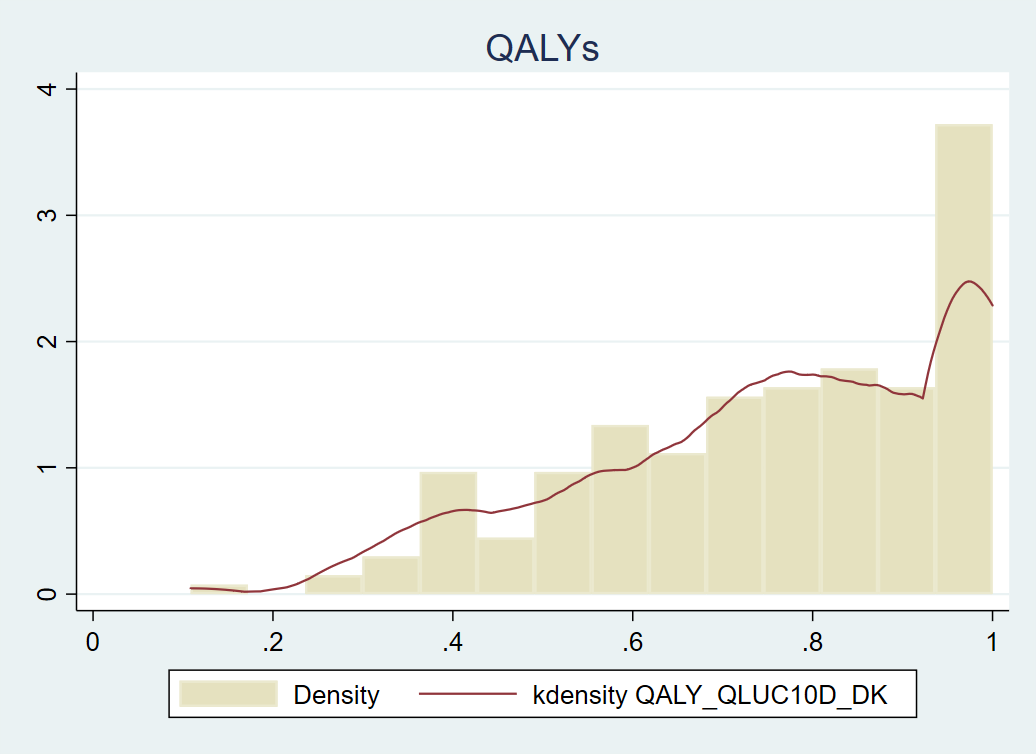
**

Figure A10. Distribution of QALYs after imputation and after matching by treatment group

**
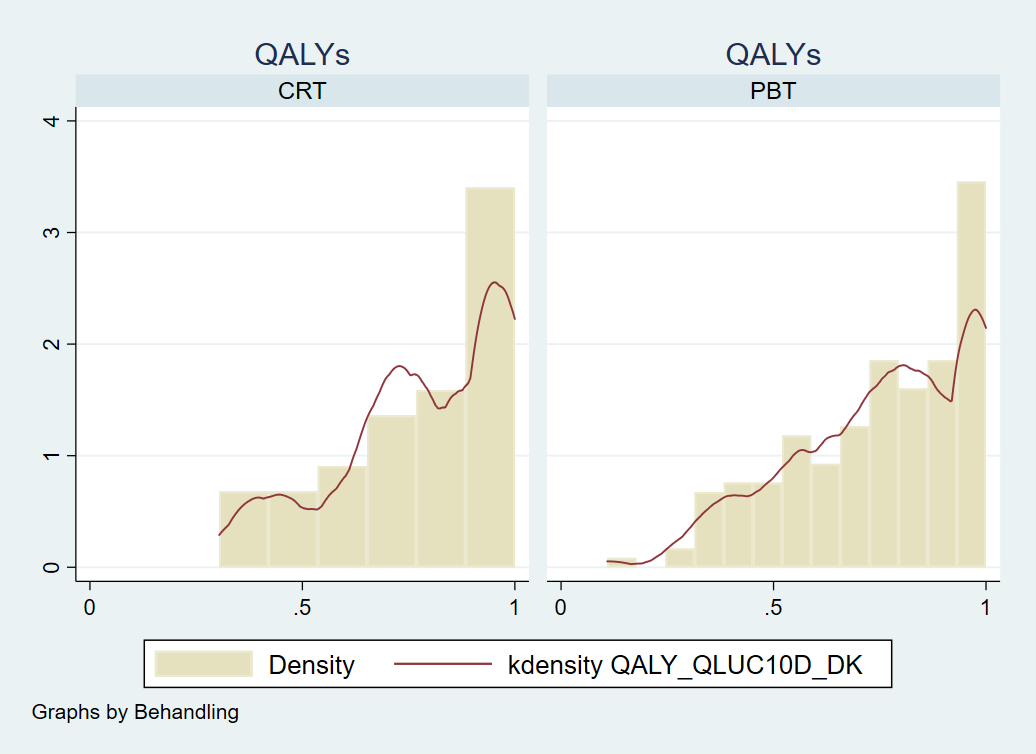
**

# Family distributions and link functions chosen for analysis of cost and QALY data

Table A4. Family distributions and link functions chosen for analysis of cost and QALY data

| Variable | Model | Family distribution | Link function |
| --- | --- | --- | --- |
| Total QALYs | GLM | Binomial | Logit |
| Total costs | GLM | Inverse gaussian | Log |
| Inpatient care costs | Two-part model* | Gamma* | Log* |
| Outpatient care costs | GLM | Gaussian | Identity |
| Medication costs | Two-part model* | Inverse gaussian* | Log* |

*Family distribution and link function chosen for the second part of the two-part model, ie. GLM

# Distribution of scores of EORTC QLQ-C30 and QLU-C10D over the study period by treatment group (after imputation and after matching)

Figure A11. Distribution of EORTC QLQ-C30 scores by treatment group

Figure A12. Distribution of QLU-C10D scores by treatment group
